# Supplementary material for: Modeling glioblastoma heterogeneity as a dynamic network of cell states
Source: Mol Syst Biol. 2021 Sep 16;17(9):e10105. doi: 10.15252/msb.202010105 (PMC8444284; doi:10.15252/msb.202010105)
Supplement: Supplementary file 5 — Source Data for Figure 3 [file MSB-17-e10105-s001.zip › Figure3A_sourcedata/GSEA_3065/hallmarks_state4.GseaPreranked.1623416296068/HALLMARK_DNA_REPAIR.html]

Details for gene set HALLMARK\_DNA\_REPAIR[GSEA]

|  || Dataset | state4 |
| Phenotype | NoPhenotypeAvailable |
| Upregulated in class | na\_neg |
| GeneSet | HALLMARK\_DNA\_REPAIR |
| Enrichment Score (ES) | -0.34897697 |
| Normalized Enrichment Score (NES) | -1.2553992 |
| Nominal p-value | 0.10193548 |
| FDR q-value | 0.2494417 |
| FWER p-Value | 0.989 |
Table: GSEA Results Summary

  

Fig 1: Enrichment plot: HALLMARK\_DNA\_REPAIR      
 Profile of the Running ES Score & Positions of GeneSet Members on the Rank Ordered List

  

| PROBE | GENE SYMBOL | GENE\_TITLE | RANK IN GENE LIST | RANK METRIC SCORE | RUNNING ES | CORE ENRICHMENT || 1 | GUK1 |  |  | 126 | 0.236 | 0.0004 | No |
| 2 | GPX4 |  |  | 183 | 0.210 | 0.0098 | No |
| 3 | SDCBP |  |  | 212 | 0.201 | 0.0230 | No |
| 4 | VPS28 |  |  | 219 | 0.196 | 0.0393 | No |
| 5 | CETN2 |  |  | 261 | 0.183 | 0.0488 | No |
| 6 | EDF1 |  |  | 393 | 0.155 | 0.0412 | No |
| 7 | POLR2J |  |  | 401 | 0.151 | 0.0534 | No |
| 8 | GTF2H5 |  |  | 404 | 0.151 | 0.0663 | No |
| 9 | SUPT4H1 |  |  | 421 | 0.148 | 0.0768 | No |
| 10 | NME4 |  |  | 432 | 0.146 | 0.0881 | No |
| 11 | TAF10 |  |  | 463 | 0.142 | 0.0958 | No |
| 12 | NME3 |  |  | 474 | 0.141 | 0.1065 | No |
| 13 | APRT |  |  | 559 | 0.130 | 0.1044 | No |
| 14 | SURF1 |  |  | 577 | 0.128 | 0.1130 | No |
| 15 | POLR3GL |  |  | 652 | 0.120 | 0.1115 | No |
| 16 | EIF1B |  |  | 656 | 0.119 | 0.1216 | No |
| 17 | POLR2I |  |  | 658 | 0.119 | 0.1319 | No |
| 18 | POLR2F |  |  | 700 | 0.114 | 0.1353 | No |
| 19 | MPG |  |  | 734 | 0.110 | 0.1396 | No |
| 20 | USP11 |  |  | 842 | 0.100 | 0.1311 | No |
| 21 | POLR2G |  |  | 912 | 0.095 | 0.1283 | No |
| 22 | RRM2B |  |  | 932 | 0.093 | 0.1334 | No |
| 23 | POLE4 |  |  | 1010 | 0.088 | 0.1286 | No |
| 24 | RBX1 |  |  | 1022 | 0.086 | 0.1345 | No |
| 25 | SNAPC5 |  |  | 1062 | 0.084 | 0.1355 | No |
| 26 | SMAD5 |  |  | 1186 | 0.077 | 0.1224 | No |
| 27 | TAF13 |  |  | 1261 | 0.073 | 0.1168 | No |
| 28 | MRPL40 |  |  | 1262 | 0.073 | 0.1232 | No |
| 29 | POLR2K |  |  | 1680 | 0.056 | 0.0604 | No |
| 30 | DCTN4 |  |  | 1702 | 0.055 | 0.0619 | No |
| 31 | NCBP2 |  |  | 1727 | 0.055 | 0.0628 | No |
| 32 | ITPA |  |  | 1745 | 0.054 | 0.0648 | No |
| 33 | VPS37D |  |  | 1747 | 0.054 | 0.0694 | No |
| 34 | TAF6 |  |  | 1844 | 0.051 | 0.0583 | No |
| 35 | GSDME |  |  | 1855 | 0.051 | 0.0612 | No |
| 36 | NELFE |  |  | 1862 | 0.050 | 0.0646 | No |
| 37 | TSG101 |  |  | 1867 | 0.050 | 0.0684 | No |
| 38 | GTF2B |  |  | 2125 | 0.043 | 0.0306 | No |
| 39 | GMPR2 |  |  | 2244 | 0.040 | 0.0150 | No |
| 40 | RALA |  |  | 2313 | 0.039 | 0.0074 | No |
| 41 | POLR1D |  |  | 2368 | 0.038 | 0.0020 | No |
| 42 | VPS37B |  |  | 2486 | 0.035 | -0.0139 | No |
| 43 | AK3 |  |  | 2544 | 0.034 | -0.0202 | No |
| 44 | POLR2E |  |  | 2630 | 0.032 | -0.0311 | No |
| 45 | DDB1 |  |  | 2721 | 0.030 | -0.0431 | No |
| 46 | SUPT5H |  |  | 2795 | 0.029 | -0.0524 | No |
| 47 | ERCC8 |  |  | 2946 | 0.026 | -0.0744 | No |
| 48 | TAF9 |  |  | 2960 | 0.025 | -0.0743 | No |
| 49 | XPC |  |  | 2998 | 0.025 | -0.0781 | No |
| 50 | GTF2F1 |  |  | 3007 | 0.025 | -0.0772 | No |
| 51 | ERCC2 |  |  | 3033 | 0.024 | -0.0792 | No |
| 52 | DGUOK |  |  | 3108 | 0.023 | -0.0891 | No |
| 53 | POLL |  |  | 3312 | 0.018 | -0.1205 | No |
| 54 | GTF2H3 |  |  | 3313 | 0.018 | -0.1189 | No |
| 55 | GTF2A2 |  |  | 3415 | 0.015 | -0.1339 | No |
| 56 | ERCC1 |  |  | 3438 | 0.014 | -0.1362 | No |
| 57 | COX17 |  |  | 3749 | 0.003 | -0.1862 | No |
| 58 | BCAM |  |  | 3797 | 0.001 | -0.1938 | No |
| 59 | PDE4B |  |  | 4063 | -0.013 | -0.2357 | No |
| 60 | MPC2 |  |  | 4412 | -0.037 | -0.2889 | No |
| 61 | RNMT |  |  | 4452 | -0.040 | -0.2916 | No |
| 62 | REV3L |  |  | 4650 | -0.055 | -0.3187 | No |
| 63 | NUDT9 |  |  | 4655 | -0.056 | -0.3145 | No |
| 64 | AAAS |  |  | 4684 | -0.058 | -0.3138 | No |
| 65 | ADRM1 |  |  | 4687 | -0.059 | -0.3090 | No |
| 66 | ADCY6 |  |  | 4818 | -0.069 | -0.3240 | No |
| 67 | IMPDH2 |  |  | 4827 | -0.070 | -0.3191 | No |
| 68 | POLD1 |  |  | 4853 | -0.072 | -0.3168 | No |
| 69 | NT5C |  |  | 4884 | -0.074 | -0.3152 | No |
| 70 | TMED2 |  |  | 4925 | -0.077 | -0.3149 | No |
| 71 | POLA1 |  |  | 5058 | -0.088 | -0.3286 | No |
| 72 | NME1 |  |  | 5086 | -0.090 | -0.3250 | No |
| 73 | POLR2H |  |  | 5127 | -0.094 | -0.3232 | No |
| 74 | DAD1 |  |  | 5287 | -0.109 | -0.3394 | Yes |
| 75 | POM121 |  |  | 5294 | -0.109 | -0.3308 | Yes |
| 76 | SEC61A1 |  |  | 5311 | -0.110 | -0.3237 | Yes |
| 77 | RPA2 |  |  | 5372 | -0.115 | -0.3232 | Yes |
| 78 | RFC5 |  |  | 5434 | -0.122 | -0.3223 | Yes |
| 79 | RFC3 |  |  | 5450 | -0.124 | -0.3138 | Yes |
| 80 | BCAP31 |  |  | 5477 | -0.129 | -0.3067 | Yes |
| 81 | NT5C3A |  |  | 5540 | -0.135 | -0.3048 | Yes |
| 82 | HPRT1 |  |  | 5554 | -0.136 | -0.2949 | Yes |
| 83 | NELFCD |  |  | 5614 | -0.146 | -0.2916 | Yes |
| 84 | POLA2 |  |  | 5615 | -0.146 | -0.2787 | Yes |
| 85 | PRIM1 |  |  | 5636 | -0.150 | -0.2687 | Yes |
| 86 | DGCR8 |  |  | 5732 | -0.168 | -0.2693 | Yes |
| 87 | SAC3D1 |  |  | 5810 | -0.186 | -0.2654 | Yes |
| 88 | ALYREF |  |  | 5955 | -0.225 | -0.2690 | Yes |
| 89 | POLD3 |  |  | 5957 | -0.225 | -0.2493 | Yes |
| 90 | LIG1 |  |  | 6002 | -0.239 | -0.2353 | Yes |
| 91 | RPA3 |  |  | 6014 | -0.244 | -0.2156 | Yes |
| 92 | FEN1 |  |  | 6035 | -0.256 | -0.1962 | Yes |
| 93 | RFC2 |  |  | 6095 | -0.294 | -0.1799 | Yes |
| 94 | DUT |  |  | 6107 | -0.303 | -0.1550 | Yes |
| 95 | ARL6IP1 |  |  | 6145 | -0.336 | -0.1313 | Yes |
| 96 | PCNA |  |  | 6173 | -0.374 | -0.1028 | Yes |
| 97 | RFC4 |  |  | 6185 | -0.399 | -0.0693 | Yes |
| 98 | ZWINT |  |  | 6195 | -0.425 | -0.0333 | Yes |
| 99 | TYMS |  |  | 6222 | -0.501 | 0.0067 | Yes |
Table: GSEA details [plain text format]

  

Fig 2: HALLMARK\_DNA\_REPAIR: Random ES distribution      
 Gene set null distribution of ES for **HALLMARK\_DNA\_REPAIR**
